# Anti–*Helicobacter pylori* Treatment in Patients With Gastric Cancer After Radical Gastrectomy

**DOI:** 10.1001/jamanetworkopen.2024.3812

**Published:** 2024-03-28

**Authors:** Zhoukai Zhao, Ruopeng Zhang, Guoming Chen, Man Nie, Feiyang Zhang, Xiaojiang Chen, Jun Lin, Zewei Chen, Feizhi Lin, Chengzhi Wei, Ziqi Zheng, Shenghang Ruan, Bowen Huang, Yingbo Chen, Runcong Nie

**Affiliations:** 1 Department of Gastric Surgery, State Key Laboratory of Oncology in South China, Guangdong Provincial Clinical Research Center for Cancer, Sun Yat-sen University Cancer Center, Guangzhou, China; 2 Department of Medical Oncology, State Key Laboratory of Oncology in South China, Guangdong Provincial Clinical Research Center for Cancer, Collaborative Innovation Center for Cancer Medicine, Sun Yat-sen University Cancer Center, Guangzhou, China

## Abstract

**Importance:**

Whether anti–*Helicobacter pylori* treatment can provide survival benefits for patients with gastric cancer who are diagnosed with *H pylori* infection is an area with limited research.

**Objective:**

To explore the potential survival benefits of anti–*H pylori* treatment after radical gastrectomy in patients with gastric cancer and presurgical confirmation of *H pylori* infection.

**Design, Setting, and Participants:**

This retrospective cohort study was conducted using data from patients with gastric cancer treated between January 1, 2010, and December 31, 2018, and followed up for outcome ascertainment until May 19, 2021. Propensity score matching was performed in patients treated with or without anti–*H pylori* treatment. This study involved a single institute in a comprehensive cancer treatment and research center located in Guangzhou, Guangdong Province, China. The study included patients with gastric or esophagogastric junction adenocarcinoma who underwent curative gastrectomy with D2 lymphadenectomy and tested positive for *H pylori* infection. Data were analyzed from March to June 2023.

**Exposure:**

Anti–*H pylori* treatment, which primarily includes triple therapy regimens consisting of amoxicillin, clarithromycin, and omeprazole for 14 days.

**Main Outcomes and Measures:**

Clinical outcomes, including overall survival (OS) and disease-free survival (DFS), were analyzed by Kaplan-Meier method, log-rank test, and Cox proportional hazards regression model. Subgroup analysis based on crucial clinical information was also conducted.

**Results:**

All 1293 patients (median [IQR] age, 59 [50-65] years; 860 [66.5%] male) were divided into 2 groups, with 125 patients in the anti–*H pylori* treatment group and 1168 patients in the non–anti–*H pylori* treatment group based on whether they received anti–*H pylori* treatment during the perioperative period and the follow-up. Survival analysis showed that the 5-year OS rates were 94.1% (95% CI, 89.3%-99.2%) in the anti–*H pylori* group and 73.8% (95% CI, 70.7%-77.0%) in the non–anti–*H pylori* group, and the hazard ratio (HR) of these 2 groups was 0.33 (95% CI, 0.18-0.60; *P* < .001). The survival benefit remained after propensity score matching (HR, 0.50; 95% CI, 0.26-0.99; *P* = .048). Multivariable analysis for OS and DFS further showed the survival benefit of anti–*H pylori* treatment, with HRs of 0.38 (95% CI, 0.17-0.87; *P* = .02) and 0.48 (95% CI, 0.28-0.83; *P* = .008), respectively. Among patients with TNM stage II/III disease who received adjuvant chemotherapy, anti–*H pylori* treatment was associated with survival benefits (OS: HR, 0.49; 95% CI, 0.24-0.99; *P* = .046), whereas among those who did not receive adjuvant chemotherapy, anti–*H pylori* treatment was not associated with survival benefits (OS: HR, 0.29; 95% CI, 0.04-2.08; *P* = .22).

**Conclusions and Relevance:**

This cohort study indicates that anti–*H pylori* treatment may be associated with improved survival in patients with gastric cancer who have *H pylori* infections. The study reinforces the importance of including *H pylori* screening and treatment in the surgical treatment of these patients.

## Key Points

**Question:**

Is anti–*Helicobacter pylori* treatment associated with enhanced postgastrectomy survival prospects for patients diagnosed with gastric cancer who have a preoperative confirmation of *H pylori* infection?

**Findings:**

In this cohort study of 1293 patients, the anti–*H pylori* treatment group had a significant survival advantage compared with the non–anti–*H pylori* treatment group in terms of overall survival and disease-free survival. After propensity score matching, the survival advantage for both overall and disease-free survival remained.

**Meaning:**

The findings indicated that for patients with gastric cancer who have *H pylori* infections, undergoing anti–*H pylori* treatment may lead to improved survival outcomes.

## Teaser

This cohort study of patients with gastric cancer in China examines the survival benefit associated with anti–*Helicobacter pylori* treatment after radical gastrectomy.

## Introduction

Despite a recent decrease in incidence in the past few years, gastric cancer (GC) continues to be a significant global health concern. It ranks as the fifth most common cancer and stands as the fourth leading cause of cancer-related deaths worldwide, accounting for more than 1.08 million new cases and 0.76 million deaths in 2020.^1^ It has been acknowledged that *Helicobacter pylori* infection is a major cause of chronic gastritis, peptic ulcer, and GC.^2^ It is estimated that 4.4 billion people worldwide are infected with *H pylori*. Similar to the incidence of GC, the rate of *H pylori* infection tends to be higher in East Asian populations.^3^

Numerous studies have provided substantial evidence that eradicating *H pylori* in healthy individuals can reduce the incidence of precancerous lesions for GC, subsequently decreasing the likelihood of developing GC.^4,5,6,7,8^ In addition, several studies have reported that patients with *H pylori–*positive GC have better survival outcomes after GC surgery compared with those with *H pylori–*negative GC.^9,10,11,12^ *Helicobacter pylori–*negative status is identified as an independent prognostic factor for poor outcomes.^10,11,12^ Although several high quality randomized clinical trials (RCTs) demonstrated that eradication of *H pylori* after endoscopic resection of early GC can significantly reduce the incidence of the development of metachronous gastric carcinoma (MGC),^13,14^ the effect of *H pylori* eradication in patients after gastrectomy have not yet been clarified. Two previous observational studies from Korea reported conflicting results in this setting. Kim et al^15^ found no significant difference in overall survival (OS), GC-specific death, and cancer recurrence rates between the anti–*H pylori* treatment group and the placebo group in patients with GC after distal gastrectomy. However, Choi et al^16^ observed a statistically significant advantage in OS and GC-specific survival for the eradication group compared with the noneradication group, with *H pylori* positivity identified as an independent risk factor for GC-specific death. The relationship between *H pylori* infection and survival rates with GC is not yet fully elucidated, limited by contradictory results, small sample size, and a paucity of high-quality related research. Thus, our study seeks to provide a deeper understanding of how anti–*H pylori* treatment influences postoperative survival outcomes in a larger, more diverse group of patients with GC, enhancing the scope of existing research findings. We conducted a retrospective cohort study from a high-volume institution to further explore the survival benefit of anti–*H pylori* treatment in patients with *H pylori*–positive GC after radical gastrectomy.

## Methods

### Patients

Retrospective collection of medical data was conducted for patients who underwent curative surgical treatment for GC at Sun Yat-sen University Cancer Center (Guangzhou, China) between January 1, 2010, and December 31, 2018. Patients were included in this cohort study if they were pathologically diagnosed as having gastric adenocarcinoma or adenocarcinoma of the esophagogastric junction, underwent curative gastrectomy with D2 lymphadenectomy, and had confirmed *H pylori* infection. Patients were excluded if they had other malignant tumors, were pathologically diagnosed with stage IV disease, underwent R1/2 resection, had uncertain or negative *H pylori* status, or had previously received anti–*H pylori* treatment within 3 months before the diagnosis of GC. Pathological staging, including depth of tumor invasion, lymph node involvement, and resection status, was evaluated according to the eighth edition of the American Joint Committee on Cancer’s *Cancer Staging Manual*.^17^

**Figure 1.**
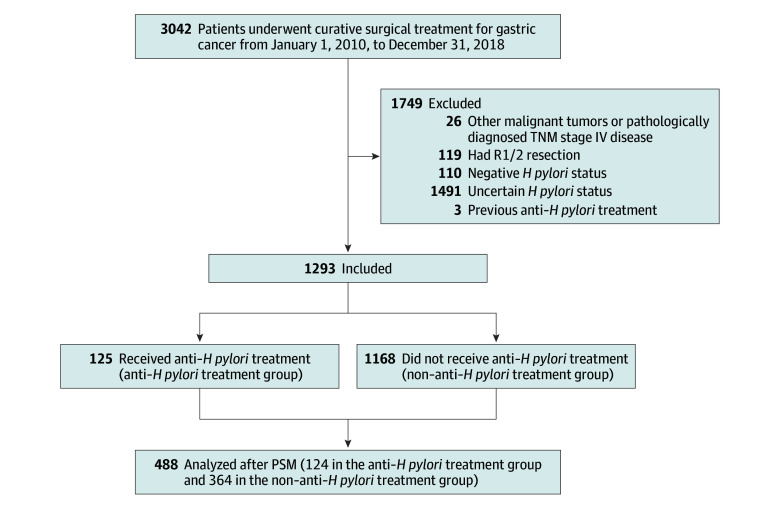
Flowchart of Patient Inclusion and Exclusion Patients who were pathologically diagnosed as having gastric adenocarcinoma or adenocarcinoma of the esophagogastric junction, underwent curative gastrectomy with D2 lymphadenectomy, and had confirmed *Helicobacter pylori* infection from January 1, 2010, to December 31, 2018, were enrolled in the study. Propensity score matching (PSM) was performed based on initially imbalanced variables.

A total of 1293 patients who met the above criteria were included in the study (Figure 1). Patients were grouped into the anti–*H pylori* treatment group (125 cases) and the non–anti–*H pylori* treatment group (1168 cases) based on whether they received anti–*H pylori* treatment during the perioperative period and the follow-up. The following demographic and clinicopathologic characteristics of these patients were recorded: age, sex, comorbidities (including hypertension, coronary heart disease, and diabetes), body mass index, history of smoking, histologic grade, Borrmann classification, Lauren classification, tumor depth of invasion, lymph node metastasis, TNM staging, maximum tumor diameter, type of gastrectomy, postoperative complications, preoperative carcinoembryonic antigen level, and adjuvant chemotherapy. Written informed consent to use the samples for research purposes was obtained from all the patients before surgery. This study was performed in accordance with the Declaration of Helsinki^18^ and was approved by the institutional review board at the Sun Yat-sen University Cancer Center. The study followed the Strengthening the Reporting of Observational Studies in Epidemiology (STROBE) reporting guideline.

### Evaluation of *H pylori* Infection Status

All included patients in the study underwent histologic examination with the Giemsa staining method, 13C urea breath test, and/or rapid urease test to ascertain their *H pylori* infection status before the gastrectomy. Patients who tested negative for *H pylori* infection before the gastrectomy were categorized as *H pylori* negative and were consequently excluded from the analysis, whereas those with 1 or more positive results were confirmed to be infected with *H pylori*.

### Anti–*H pylori* Treatment

According to the World Gastroenterology Organization global *H pylori* guidelines, the first-line treatment includes triple or quadruple therapy regimens, and the recommended treatment duration is 7 to 14 days.^19^ In clinical practice, variations in antibiotic selection may occur based on patients’ condition and physicians’ preferences. During data collection, 2 independent investigators carefully reviewed the recorded medications of each patient and confirmed whether the patients received an appropriate anti–*H pylori* treatment. According to the data collected retrospectively, the patients in our institution primarily received a triple anti–*H pylori* therapy consisting of amoxicillin, clarithromycin, and omeprazole for 14 days, and they were often advised by clinicians to start anti–*H pylori* treatment during their postoperative follow-up, particularly 4 to 6 weeks after gastrectomy, when a positive *H pylori* test result was observed.

### Patient Follow-Up

Patients received regular follow-up care through outpatient or inpatient visits or through telephone calls every 3 to 6 months during the initial 2 years after surgery, every 6 to 12 months during the 3 to 5 years after surgery, and annually thereafter. In this study, the median (range) follow-up time was 40.4 (0.2-131.3) months. The main follow-up examinations included physical examinations (eg, palpation of left supraclavicular lymph node and digital rectal examination), tumor marker measurement, computed tomography examination, ultrasonographic examination, and endoscopy.

### Outcomes

In this study, the primary end point was OS, defined as the time from the date of surgery to the date of death from any cause. The secondary end point was disease-free survival (DFS), defined as the time from the date of surgery to disease progression, relapse, or death, whichever came first. The follow-up period for outcome ascertainment in our study extended until May 19, 2021.

### Statistical Analysis

The χ^2^ or Fisher test was used to compare differences between groups for categorical variables. Propensity score matching (PSM) was conducted to minimize the selection biases between the 2 groups in the overall cohort, with a ratio of 1:3 and a caliper value of 0.02, using the nearest matching method. The variables used for PSM included Borrmann classification, pathological T and N stages, TNM stage, tumor maximum diameter, type of gastrectomy, and adjuvant chemotherapy. This selection focused on variables that were initially imbalanced or potentially related to clinical outcomes. Kaplan-Meier analysis was used to generate the survival curves, and log-rank tests were used to compare the survival differences between groups. To explore the potential survival benefits of anti–*H pylori* treatment across specific patient populations, we conducted exploratory subgroup analyses based on the majority of clinical information collected, such as sex, age, and TNM staging, while taking into account the sample size. Then, in the overall cohort, the variables with statistical significance in the univariable analysis were entered into the multivariable Cox proportional hazards regression analysis for both OS and DFS. All statistical analyses were 2-sided, with *P* < .05 considered statistically significant. Data analysis and graphical visualization were performed using R software, version 4.2.2 (R Foundation for Statistical Computing). Data were analyzed from March to June 2023.

## Results

### Patient Characteristics

In this study, 1293 patients (median [IQR] age, 59 [50-65] years; 860 [66.5%] male and 433 [33.5%] female) with preoperatively confirmed *H pylori* infection were included. These participants had a median (IQR) body mass index of 22.0 (19.6-24.0) (calculated as weight in kilograms divided by height in meters squared), and 829 (64.1%) underwent distal gastrectomy. Postoperatively, 600 patients (46.4%) were pathologically diagnosed with TNM stage III disease and 808 (62.5%) received adjuvant chemotherapy. However, as indicated in the Table, only 125 (9.7%) had received anti–*H pylori* treatment. There were no statistically significant differences between the anti–*H pylori* treatment group and the non–anti–*H pylori* treatment group in terms of sex, age, body mass index, smoking history, and comorbidities. However, the anti–*H pylori* treatment group had a higher proportion of distal gastrectomy (102 of 125 [81.6%] vs 727 of 1168 [62.2%]; *P* < .001) and received less adjuvant chemotherapy (54 of 125 [43.2%] vs 754 of 1168 [64.6%]; *P* < .001) compared with the non–anti–*H pylori* treatment group. Of note, patients in the non–anti–*H pylori* treatment group had advanced pathological T stage, N stage, and TNM stage as well as larger tumor maximum diameter, which indicated the potential selection bias between the 2 groups.

**Table.** Patient Demographic, Clinical, and Pathological Characteristics Stratified by Anti–*Helicobacter pylori* Treatment Before and After PSM

Variable	Anti–*H pylori* treatment					
	Before PSM			After PSM		
	Yes, No. (%) (n = 125)	No, No. (%) (n = 1168)	*P* value^a^	Yes, No. (%) (n = 124)	No, No. (%) (n = 364)	*P* value^a^
Sex						
Male	85 (68.0)	775 (66.4)	.71	84 (67.7)	240 (65.9)	.71
Female	40 (32.0)	393 (33.6)		40 (32.3)	124 (34.1)	
Age group, y						
<60	75 (60.0)	622 (53.3)	.15	75 (60.5)	215 (59.1)	.78
≥60	50 (40.0)	546 (46.7)		49 (39.5)	149 (40.9)	
Classification of BMI						
Normal weight (18.5-24)	78 (62.4)	700 (59.9)	.59	77 (62.1)	230 (63.2)	.83
Abnormal weight (<18.5 or ≥24)	47 (37.6)	468 (40.1)		47 (37.9)	134 (36.8)	
History of smoking						
No	81 (64.8)	743 (63.6)	.79	81 (65.3)	232 (63.7)	.75
Yes	44 (35.2)	425 (36.4)		43 (34.7)	132 (36.3)	
Hypertension						
No	109 (87.2)	1005 (86.0)	.72	108 (87.1)	315 (86.5)	.87
Yes	16 (12.8)	163 (14.0)		16 (12.9)	49 (13.5)	
Coronary heart disease						
No	124 (99.2)	1155 (98.9)	>.99	123 (99.2)	359 (98.6)	>.99
Yes	1 (0.8)	13 (1.1)		1 (0.8)	5 (1.4)	
Diabetes						
No	119 (95.2)	1086 (93.0)	.35	118 (95.2)	342 (94.0)	.62
Yes	6 (4.8)	82 (7.0)		6 (4.8)	22 (6.0)	
Histologic grade						
G1/G2	48 (38.4)	413 (35.4)	.77	48 (38.7)	142 (39.0)	.84
G3/G4	69 (55.2)	683 (58.5)		68 (54.8)	193 (53.0)	
Others	8 (6.4)	72 (6.2)		8 (6.5)	29 (8.0)	
Borrmann classification						
I-III	116 (92.8)	1117 (95.6)	.10	116 (93.5)	349 (95.9)	.14
IV	4 (3.2)	34 (2.9)		4 (3.2)	12 (3.3)	
Unknown	5 (4.0)	17 (1.5)		4 (3.2)	3 (0.8)	
Lauren classification						
Diffused	50 (40.0)	445 (38.1)	.80	50 (40.3)	142 (39.0)	.56
Intestinal	31 (24.8)	328 (28.1)		30 (24.2)	110 (30.2)	
Mixed	28 (22.4)	270 (23.1)		28 (22.6)	67(18.4)	
Unknown	16 (12.8)	125 (10.7)		16 (12.9)	45 (12.4)	
Pathological T stage						
T1/2	82 (65.6)	382 (32.7)	<.001	81 (65.3)	233 (64.0)	.79
T3/4	43 (34.4)	786 (67.3)		43 (34.7)	131 (36.0)	
Pathological N stage						
N (−)	75 (60.0)	396.(33.9)	<.001	74 (59.7)	213 (58.5)	.82
N (+)	50 (40.0)	772.0 (66.1)		50 (40.3)	151 (41.5)	
TNM stage						
I	70 (56.0)	280 (24.0)	<.001	69 (55.6)	199 (54.7)	.85
II/III	55 (44.0)	888 (76.0)		55 (44.4)	165 (45.3)	
Tumor maximum diameter, cm						
≤4	99 (79.2)	621 (53.2)	<.001	99 (79.8)	294 (80.8)	.82
>4	26 (20.8)	547 (46.8)		25 (20.2)	70 (19.2)	
Type of gastrectomy						
Distal	102 (81.6)	727 (62.2)	<.001	101 (81.5)	297(81.6)	.97
Proximal	11 (8.8)	129 (11.0)		11 (8.9)	34 (9.3)	
Total	12 (9.6)	312 (26.7)		12 (9.7)	33 (9.1)	
CEA, ng/mL						
≤5	112 (89.6)	971 (83.1)	.06	112 (90.3)	326 (89.6)	.81
>5	13 (10.4)	197 (16.9)		12 (9.7)	38 (10.4)	
Adjuvant chemotherapy						
No	71 (56.8)	414 (35.4)	<.001	70 (56.5)	202 (55.5)	.85
Yes	54 (43.2)	754 (64.6)		54 (43.5)	162 (44.5)	
Postoperative complication						
No	116 (92.8)	1098 (94.0)	.59	115 (92.7)	344 (94.5)	.47
Yes	9 (7.2)	70 (6.0)		9 (7.3)	20 (5.5)	

Abbreviations: BMI, body mass index (calculated as weight in kilograms divided by height in meters squared); CEA, carcinoembryonic antigen; PSM, propensity score matching.

SI conversion factors: To convert CEA to micrograms per liter, multiply by 1.

^a^ Pearson χ^2^ test or Fisher exact test.

### Propensity Score Matching

To minimize the potential influence of selection bias, we used PSM. After PSM, a total of 124 patients in the anti–*H pylori* treatment group and 364 patients in the non–anti–*H pylori* treatment group were included, and the clinical characteristics between the 2 groups were well balanced (Table).

### Survival Analysis of Patients According to Anti–*H pylori* Treatment

**Figure 2.**
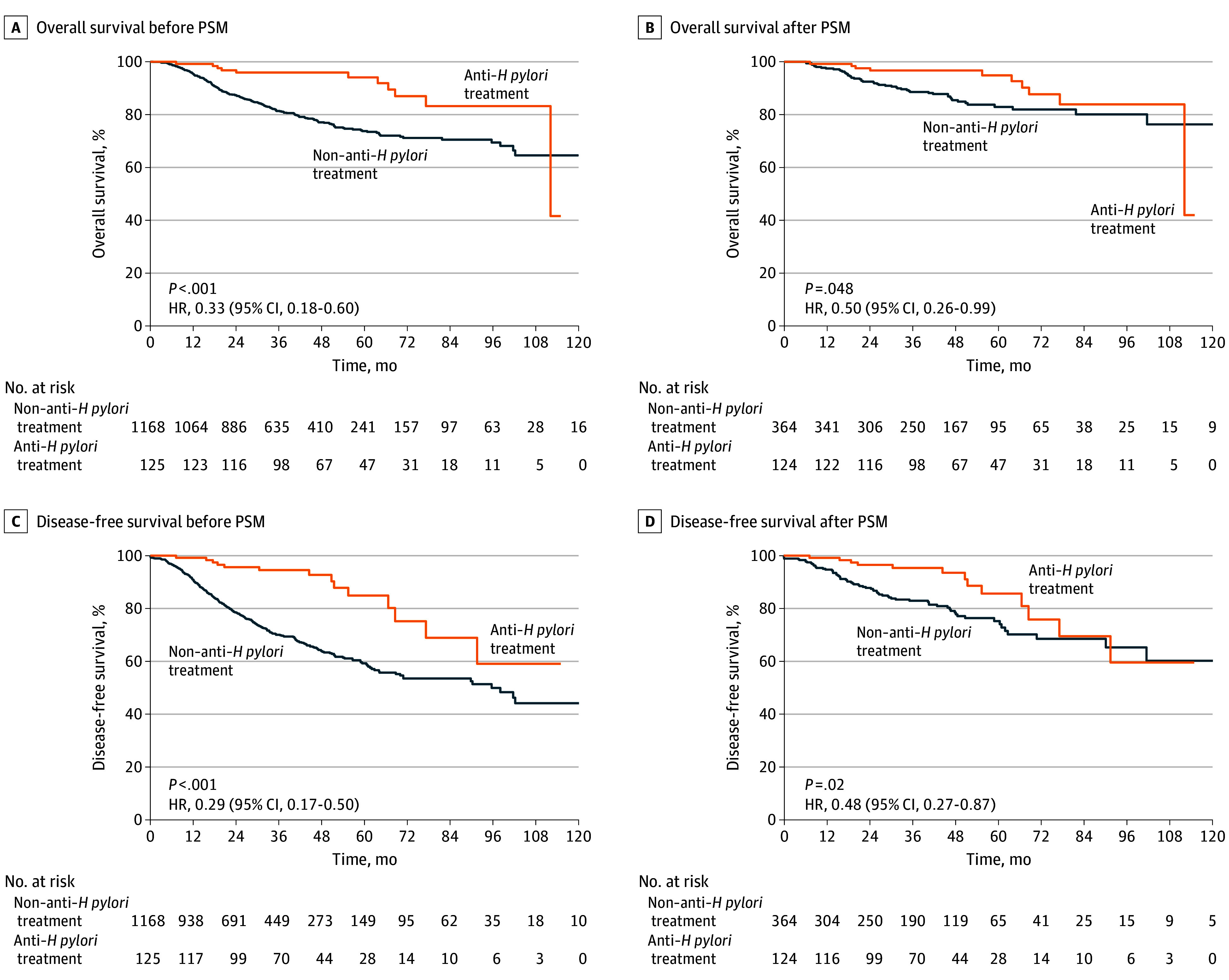
Kaplan-Meier Analysis for Overall Survival and Disease-Free Survival of the Patients With Gastric Cancer in the Anti–*Helicobacter pylori* Treatment Group Compared With Those in the Non–Anti–*H pylori* Treatment Group Before and After Propensity Score Matching (PSM) HR indicates hazard ratio.

We conducted a survival analysis to compare OS of the 2 groups (Figure 2A). The OS rates were 95.9% (95% CI, 92.5%-99.5%) at 3 years and 94.1% (95% CI, 89.3%-99.2%) at 5 years in the anti–*H pylori* treatment group compared with 81.4% (95% CI, 79.0%-83.8%) at 3 years and 73.8% (95% CI, 70.7%-77.0%) at 5 years in the non–anti–*H pylori* treatment group. The OS difference between the 2 groups was significant (hazard ratio [HR], 0.33; 95% CI, 0.18-0.60; *P* < .001). After PSM, the difference in OS between the 2 groups remained statistically significant (HR, 0.50; 95% CI, 0.26-0.99; *P* = .048) (Figure 2B).

Subsequently, survival analysis on the DFS of the 2 groups was conducted. The DFS rates were 94.5% (95% CI, 90.3%-98.9%) at 3 years and 84.9% (95% CI, 75.6%-95.4%) at 5 years in the anti–*H pylori* treatment group compared with 70.0% (95% CI, 67.1%-73.1%) at 3 years and 59.2% (95% CI, 55.4%-63.3%) at 5 years in the non–anti–*H pylori* treatment group. As shown in Figure 2C, the anti–*H pylori* treatment group also had a significant advantage in DFS compared with the non–anti–*H pylori* treatment group (HR, 0.29; 95% CI, 0.17-0.50; *P* < .001). After PSM, the survival difference in DFS remained statistically significant (HR, 0.48; 95% CI, 0.27-0.87; *P* = .02) (Figure 2D).

### Survival Analysis of Patients in Different Subgroups

**Figure 3.**
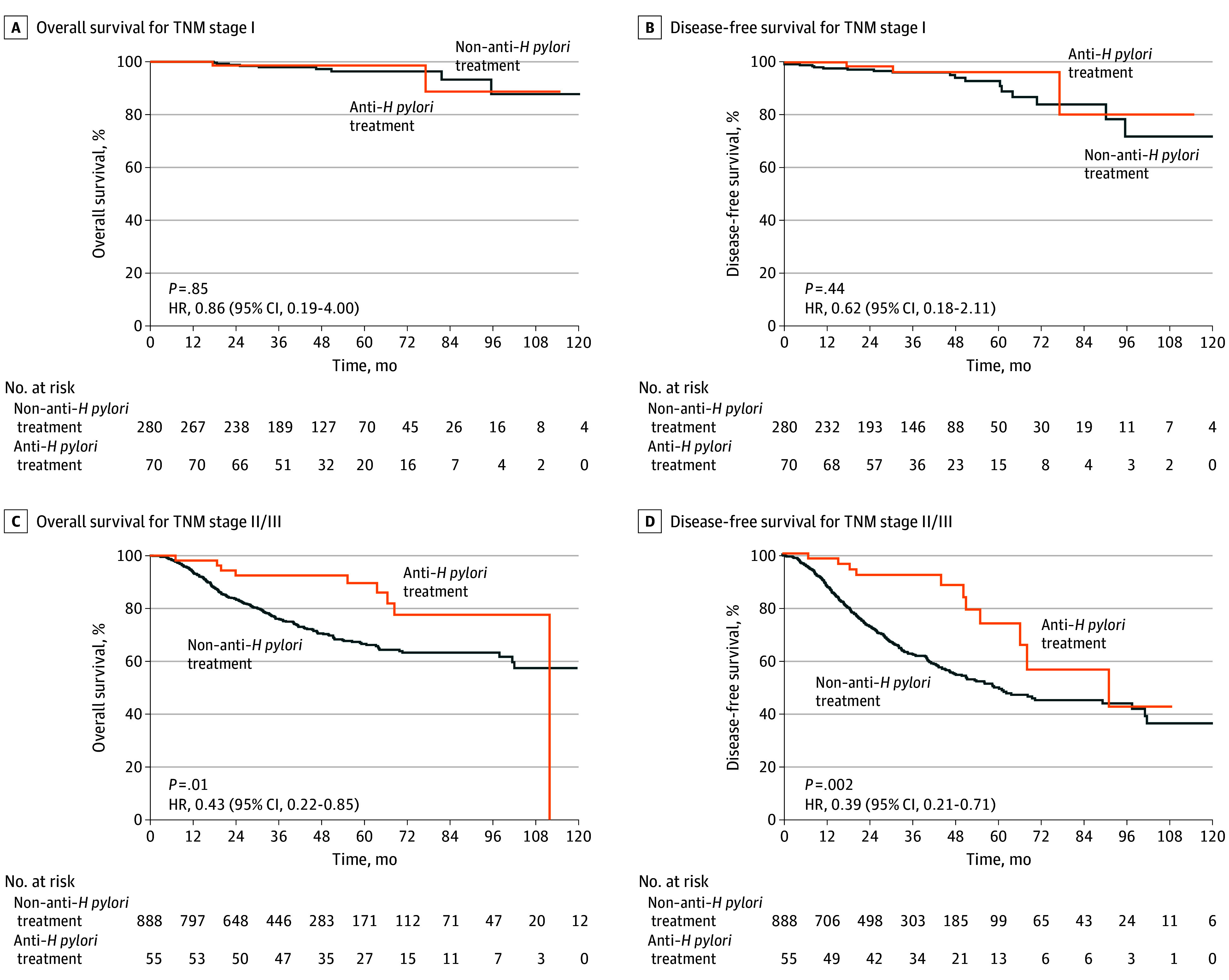
Kaplan-Meier Analysis for Overall Survival and Disease-Free Survival of Patients With Gastric Cancer in the Anti–*Helicobacter pylori* Treatment Group Compared With Those in the Non–Anti–*H pylori* Treatment Group in TNM Stage I and Stage II/III Subgroups HR indicates hazard ratio.

**Figure 4.**
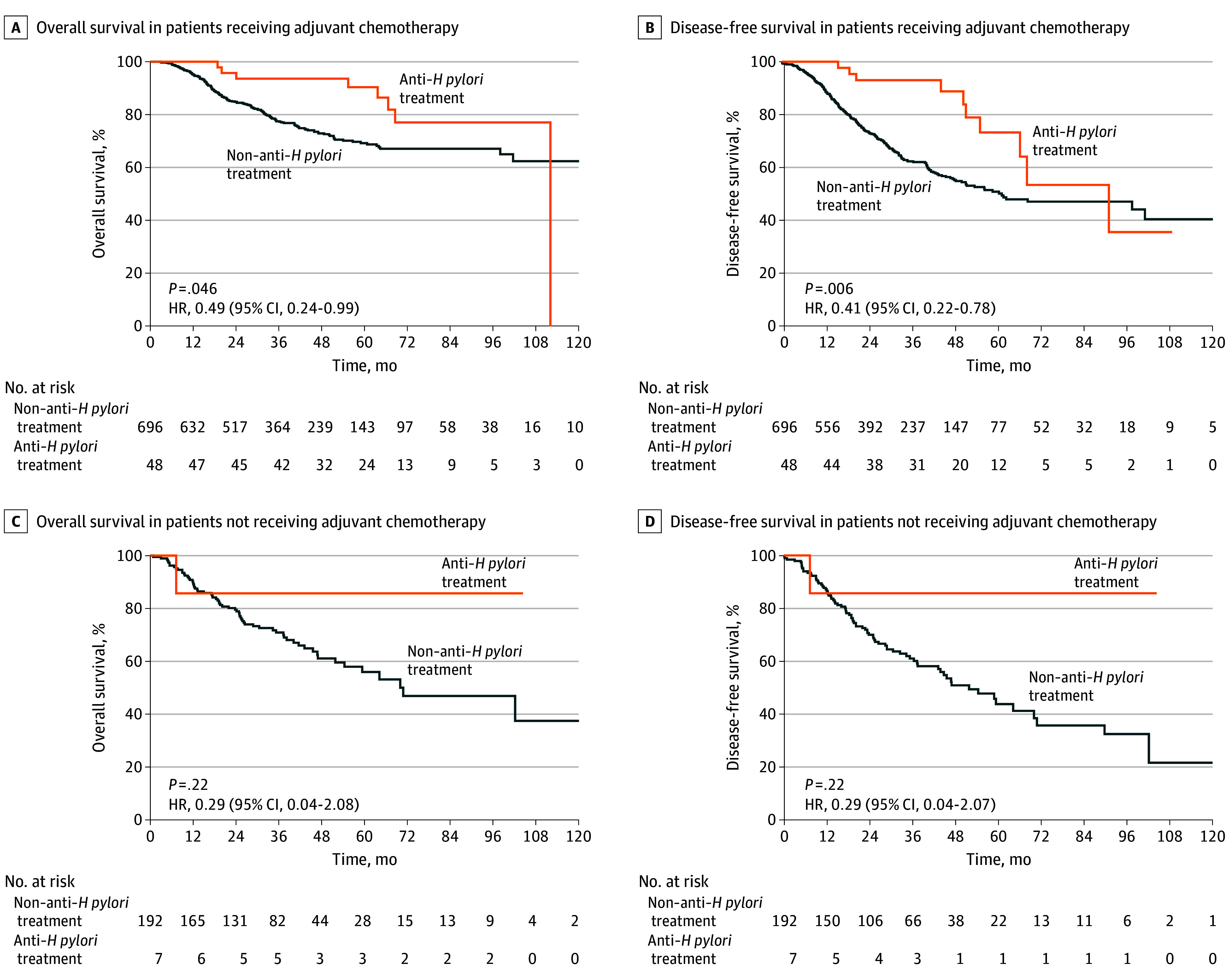
Kaplan-Meier Analysis for Overall Survival and Disease-Free Survival of the Patients With TNM Stage II/III Gastric Cancer Comparing Anti–*Helicobacter pylori* With Non–Anti–*H pylori* Treatment Groups in the Subgroups Receiving and Not Receiving Adjuvant Chemotherapy HR indicates hazard ratio.

Next, we conducted subgroup analysis of OS and DFS (eFigures 1 and 2 in Supplement 1). The relative treatment benefit of anti–*H pylori* was consistent across most of the subgroups, specifically with relation to age, sex, and tumor differentiation. In terms of TNM stage, we found that the differences in OS (HR, 0.86; 95% CI, 0.19-4.00; *P* = .85) (Figure 3A) and in DFS (HR, 0.62; 95% CI, 0.18-2.11; *P* = .44) (Figure 3B) between the anti–*H pylori* treatment group and non–anti–*H pylori* treatment group were not significant in patients with stage I disease. However, in patients with stage II/III disease, the differences in OS (HR, 0.43; 95% CI, 0.22-0.85; *P* = .01) (Figure 3C) and DFS (HR, 0.39; 95% CI, 0.21-0.71; *P* = .002) (Figure 3D) between the 2 groups were significant. We also explored the survival benefit of anti–*H pylori* treatment for patients with TNM stage II/III disease who did or did not undergo adjuvant chemotherapy. The results indicated that among patients who received adjuvant chemotherapy, anti–*H pylori* treatment conferred survival benefits for both OS (HR, 0.49; 95% CI, 0.24-0.99; *P* = .046) (Figure 4A) and DFS (HR, 0.41; 95% CI, 0.22-0.78; *P* = .006) (Figure 4B), whereas among those who did not receive adjuvant chemotherapy, anti–*H pylori* treatment did not provide survival benefits for either OS (HR, 0.29; 95% CI, 0.04-2.08; *P* = .22) (Figure 4C) or DFS (HR, 0.29; 95% CI, 0.04-2.07; *P* = .22) (Figure 4D).

### Prognostic Factor Analysis With Cox Proportional Hazards Regression Model

Furthermore, univariable and multivariable analyses using the Cox proportional hazards regression model were performed. As detailed in eTable 1 in Supplement 1, age, Lauren classification, TNM stage, tumor maximum diameter, adjuvant chemotherapy, and anti–*H pylori* treatment were associated with the OS in the univariable analysis. In the multivariable analysis, age (≥60 years [HR, 1.47; 95% CI, 1.10-1.96; *P* = .009]), Lauren classification (intestinal type [HR, 0.67; 95% CI, 0.48-0.95; *P* = .02] and mixed type [HR, 0.62; 95% CI, 0.44-0.87; *P* = .007]), TNM stage II/III (HR, 8.87; 95% CI, 3.99-19.75; *P* < .001), tumor maximum diameter (>4 cm [HR, 2.33; 95% CI, 1.72-3.18; *P* < .001]), adjuvant chemotherapy (HR, 0.63; 95% CI, 0.46-0.85; *P* = .003), and anti–*H pylori* treatment (HR, 0.38; 95% CI, 0.17-0.87; *P* = .02) were identified as independent factors associated with OS. In eTable 2 in Supplement 1, univariable and multivariable analyses for DFS showed that TNM stage II/III (HR, 4.61; 95% CI, 2.84-7.49; *P* < .001), tumor maximum diameter (>4 cm [HR, 2.37; 95% CI, 1.87-3.01; *P* < .001]), and anti–*H pylori* treatment (HR, 0.48; 95% CI, 0.28-0.83; *P* = .008) remained associated with DFS.

## Discussion

To the best of our knowledge, this is the largest study to investigate the survival benefit of anti–*H pylori* treatment in patients with *H pylori*–positive GC after radical gastrectomy. Our study found that postgastrectomy anti–*H pylori* treatment may provide substantial survival benefits in terms of both OS and DFS in a large cohort subjected to a long-term follow-up period of up to 10 years. The aforementioned conclusions remained consistent after PSM. The positive association of anti–*H pylori* treatment remained consistent across most subgroups, with significant OS and DFS benefits particularly pronounced in the TNM stage II/III subgroup. In contrast, the TNM stage I subgroup did not exhibit such benefits. Notably, although the patients with TNM stage II/III disease who received adjuvant chemotherapy derived benefits from anti–*H pylori* treatment, those who did not undergo such chemotherapy did not experience these advantages. Furthermore, Cox proportional hazards regression multivariable analysis demonstrated that anti–*H pylori* treatment is independently associated with both OS and DFS.

The worldwide prevalence of *H pylori* infection is significant. In 2015, approximately 4.4 billion individuals were infected with *H pylori*, constituting approximately 50% of the total population.^3^ Notably, *H pylori* infection increases the risk of GC by approximately 3 times and is the most significant risk factor for GC.^20^ The role of *H pylori* infection in facilitating the development of GC is well established. However, the correlation between *H pylori* infection and the prognosis of patients with GC is yet to be clearly defined. Some studies have reported that patients who test negative for *H pylori* before undergoing curative GC surgery seem to have a worse prognosis compared with *H pylori*–positive patients.^2,10,11,21^ Furthermore, a previous study^12^ indicated that *H pylori*–negative GC is associated with worse OS and has intrinsic correlations with adverse pathological and clinical characteristics.

In contrast, there is evidence suggesting that *H pylori* infection may play a role in the progression of gastric mucosal carcinogenesis in the remnant stomach after gastrectomy for GC.^22^ In the Korean guidelines for *H pylori*, eradication of *H pylori* is recommended to prevent gastric mucosal dysplasia and even GC recurrence in patients who undergo distal gastrectomy.^23^ Furthermore, several high-quality RCTs have demonstrated that prophylactic eradication of *H pylori* after endoscopic resection of early GC could prevent the development of MGC. Fukase et al^13^ reported that the odds ratio for developing MGC was 0.353 (95% CI, 0.161-0.775; *P* = .009) in the full intention-to-treat population, whereas the hazard ratio for developing MGC was 0.339 (95% CI, 0.157-0.729; *P* = .003) in the modified intention-to-treat population when comparing the eradication group with the control group. The study performed by Choi et al,^14^ which aimed to assess the long-term effects of *H pylori* eradication treatment on histologic improvement and the prevention of MGC in patients after endoscopic resection for early GC or high-grade adenoma, reported that MGC developed in 14 patients (7.2%) in the treatment group and 27 patients (13.4%) in the placebo group, with an HR of 0.50 in the treatment group (95% CI, 0.26-0.94; *P* = .03).

In the clinical setting, both the actual rate of anti–*H pylori* treatment use and the *H pylori* eradication rate remain low. In our study, few *H pylori*–positive patients (9.7%) opted for anti*–H pylori* treatment, and it remains an exploratory and unresolved question whether *H pylori* eradication is beneficial to the prognosis of patients after gastrectomy. To the best of our knowledge, only a few researchers have conducted exploratory studies in this area, and the findings are in dispute. The study conducted by Kim et al,^15^ which was a retrospective outcome event analysis extracted from a prospective cohort of an RCT study involving 169 patients, reported that among patients with GC after distal gastrectomy, there were no significant differences between the anti–*H pylori* treatment group and the placebo group in the 5-year OS, 5-year cumulative GC-specific death, and 5-year cancer recurrence rates. However, the study had certain limitations; it originated from an RCT cohort initially focused on evaluating *H pylori* eradication effects on mucosal atrophy and intestinal metaplasia in the remnant stomach after distal gastrectomy. Additionally, the study lacked patient stratification and was constrained by a relatively small sample size. In another study of 1031 patients with GC who underwent subtotal gastrectomy, Choi et al^16^ reported that the eradication group had a statistically significant advantage in terms of OS and GC-specific survival compared with the noneradication group, and this advantage persisted after PSM. However, the study included patients who tested positive for *H pylori* during the first postoperative gastroscopy follow-up, which occurred annually after the gastrectomy, and who underwent *H pylori* eradication treatment within 2 years. As a result, the interval between surgery and undergoing the test for *H pylori* infection in this study may be too long, and the infection status of *H pylori* may vary after surgery.

We designed this large-scale study to provide relevant evidence for future clinical practice for patients with GC and *H pylori* infection. We found that patients with *H pylori* infection who received anti–*H pylori* treatment experienced survival advantages in terms of OS and DFS compared with those who did not receive the treatment, and these advantages persisted even after PSM, similar to the previous study mentioned above.^16^ Furthermore, detailed stratified analyses revealed that most subgroups experienced the survival benefit of anti–*H pylori* treatment. In our Cox proportional hazard regression models, anti–*H pylori* treatment stood out as an independent prognostic factor for both OS and DFS.

Imbalances in factors related to survival between the 2 patient groups could impact the accuracy of the analysis. Therefore, it may be crucial to perform the subgroup analysis and use PSM. The previous study conducted by Choi et al^16^ found that *H pylori* eradication treatment has statistically significant survival benefits in both patients with early GC and those with advanced GC after subtotal gastrectomy. However, our subgroup analysis based on TNM stage indicated that the anti–*H pylori* treatment group had a significant advantage in both OS and DFS compared with the non–anti–*H pylori* treatment group only in patients with TNM stage II/III GC, indicating that anti–*H pylori* treatment may provide survival benefits only in patients with advanced-stage GC.

Another intriguing observation is that although anti–*H pylori* treatment offered survival benefits to the patients with TNM stage II/III who underwent adjuvant chemotherapy, no such difference was noted in those patients without adjuvant chemotherapy. This finding suggests that combining anti–*H pylori* treatment with adjuvant chemotherapy might offer enhanced benefits to patients. To further substantiate our conjecture, we also designed an RCT that aimed to corroborate whether anti–*H pylori* treatment can bring survival benefits to patients with surgically resected GC.^24^ We hope that with the emergence of more relevant research, more effective treatment strategies will be available for patients with *H pylori *infection and GC.

### Limitations

We acknowledge that the current study has several limitations. First, it is a retrospective analysis based on clinical data from a single institution, which may introduce selection bias. Second, the study period is relatively long, during which the details of surgical procedures and postoperative treatments received by patients may have varied. Third, the sample size is relatively small in certain subgroup analyses, which may result in limited statistical power.

## Conclusion

This study’s findings indicate that for patients with GC and *H pylori* infection before surgery, undergoing anti–*H pylori* treatment may be associated with notable survival advantages. We suggest expanding the scope of future *H pylori* treatment guidelines and implementing thorough screening and treatment for *H pylori* in patients undergoing surgical treatment for GC.

## Notes

Supplement 1. **eTable 1.** Univariable and Multivariable Survival Analysis of Overall Survival in the Overall Cohort (Before PSM: n = 1293) **eTable 2.** Univariable and Multivariable Survival Analysis of Disease-Free Survival in the Overall Cohort (Before PSM: n = 1293) **eFigure 1.** Subgroup Analyses for Overall Survival According to Clinical Indicators Including Age, Gender, Tumor Differentiation, Etc **eFigure 2.** Subgroup Analyses for Disease-Free Survival According to Clinical Indicators Including Age, Gender, Tumor Differentiation, Etc

Supplement 2.
Data Sharing Statement
