# Supplementary material for: Anti–Helicobacter pylori Treatment in Patients With Gastric Cancer After Radical Gastrectomy
Source: JAMA Netw Open. 2024 Mar 28;7(3):e243812. doi: 10.1001/jamanetworkopen.2024.3812 (PMC10979314; doi:10.1001/jamanetworkopen.2024.3812)
Supplement: Supplement 1. — eTable 1. Univariable and Multivariable Survival Analysis of Overall Survival in the Overall Cohort (Before PSM: n = 1293) eTable 2. Univariable and Multivariable Survival Analysis of Disease-Free Survival in the Overall Cohort (Before PSM: n = 1293) eFigure 1. Subgroup Analyses for Overall Survival According to Clinical Indicators Including Age, Gender, Tumor Differentiation, Etc eFigure 2. Subgroup Analyses for Disease-Free Survival According to Clinical Indicators Including Age, Gender, Tumor Differentiation, Etc [file jamanetwopen-e243812-s001.pdf]

Supplementary Online Content

Zhao Z, Zhang R, Chen G, et al. Anti–*Helicobacter pylori* treatment in patients with gastric cancer after radical gastrectomy. *JAMA Netw Open*. 2024;7(3):e243812. doi:10.1001/jamanetworkopen.2024.3812

eTable 1. Univariable and Multivariable Survival Analysis of Overall Survival in the Overall Cohort (Before PSM: n=1293)

eTable 2. Univariable and Multivariable Survival Analysis of Disease-Free Survival in the Overall Cohort (Before PSM: n=1293)

eFigure 1. Subgroup Analyses for Overall Survival According to Clinical Indicators Including Age Group, Sex, Tumor Differentiation, etc

eFigure 2. Subgroup Analyses for Disease-Free Survival According to Clinical Indicators Including Age Group, Sex, Tumor Differentiation, etc

This supplementary material has been provided by the authors to give readers additional information about their work.

© 2024 Zhao Z et al. *JAMA Network Open.*

eTable 1. Univariable and Multivariable Survival Analysis of Overall Survival in the Overall Cohort (before PSM: n=1293)

	Univariable Cox analysis		Multivariable Cox analysis	
**Variables**	**HR (95%CI)**	***P* value**	**HR (95%CI)**	***P* value**
**Sex**				
Male	Ref			
Female	0.80 (0.61-1.05)	0.11		
**Age group, y**				
<60	Ref		Ref	
≥60	1.51 (1.18-1.92)	**0.001**	1.47 (1.10-1.96)	**0.009**
**Classification of BMI**				
Normal weight (18.5-24)	Ref			
Abnormal weight (<18.5 or ≥24)	1.05 (0.82-1.34)	0.71		
**Hypertension**				
No	Ref			
Yes	0.91 (0.63-1.31)	0.61		
**Coronary heart disease**				
No	Ref			
Yes	1.40 (0.52-3.76)	0.51		
**Diabetes**				
No	Ref			
Yes	1.47 (0.96-2.25)	0.08		
**Lauren classification**				
Diffused	Ref		Ref	
Intestinal	0.62 (0.44-0.86)	**0.005**	0.67 (0.48-0.95)	**0.02**
Mixed	0.70 (0.50-0.99)	**<0.05**	0.62 (0.44-0.87)	**0.007**
**TNM stage**				

© 2024 Zhao Z et al. *JAMA Network Open.*

	Univariable Cox analysis		Multivariable Cox analysis	
**Variables**	**HR (95%CI)**	***P* value**	**HR (95%CI)**	***P* value**
I	Ref		Ref	
II/III	9.75 (5.33-17.84)	**<0.001**	8.87 (3.99-19.75)	**<0.001**
**Tumor maximum diameter, cm**				
≤4	Ref		Ref	
>4	3.96 (3.02-5.19)	**<0.001**	2.33 (1.72-3.18)	**<0.001**
**Adjuvant chemotherapy**				
No	Ref		Ref	
Yes	1.43 (1.09-1.86)	**0.009**	0.63 (0.46-0.85)	**0.003**
**Postoperative complication**				
No	Ref			
Yes	1.12 (0.70-1.78)	0.65		
**Anti – *H pylori* treatment**				
No	Ref		Ref	
Yes	0.33 (0.18-0.60)	**<0.001**	0.38 (0.17-0.87)	**0.02**

Abbreviations: HR: hazard ratio; BMI: Body mass index; CI: confidence interval; Ref: Reference
The variables with statistical significance in the univariable analysis were entered into the multivariable Cox regression analysis, such as Age group, Lauren classification, TNM stage, tumor maximum diameter, adjuvant chemotherapy and anti–H pylori treatment.

© 2024 Zhao Z et al. *JAMA Network Open.*

eTable 2. Univariable and Multivariable Survival Analysis of Disease-Free Survival in the Overall Cohort (before PSM: n=1293)

	Univariable Cox analysis		Multivariable Cox analysis	
**Variables**	**HR (95%CI)**	***P* value**	**HR (95%CI)**	***P* value**
**Sex**				
Male	Ref			
Female	0.89 (0.72-1.11)	0.31		
**Age group, y**				
<60	Ref		Ref	
≥60	1.39 (1.13-1.71)	**0.002**	1.18 (0.95-1.46)	0.13
**Classification of BMI**				
Normal weight (18.5-24)	Ref			
Abnormal weight (<18.5 or ≥24)	1.12 (0.91-1.38)	0.27		
**Hypertension**				
No	Ref			
Yes	0.93 (0.69-1.26)	0.64		
**Coronary heart disease**				
No	Ref			
Yes	0.87 (0.33-2.34)	0.79		
**Diabetes**				
No	Ref		Ref	
Yes	1.53 (1.07-2.17)	**0.02**	1.34 (0.94-1.91)	0.11
**Lauren classification**				
Diffused	Ref			
Intestinal	0.81 (0.62-1.07)	0.13		
Mixed	0.90 (0.68-1.19)	0.47		
**TNM stage**				

	Univariable Cox analysis		Multivariable Cox analysis	
**Variables**	**HR (95%CI)**	***P* value**	**HR (95%CI)**	***P* value**
I	Ref		Ref	
II/III	7.38 (4.75-11.47)	**<0.001**	4.61 (2.84-7.49)	**<0.001**
**Tumor maximum diameter, cm**				
≤4	Ref		Ref	
>4	3.73 (2.98-4.66)	**<0.001**	2.37 (1.87-3.01)	**<0.001**
**Adjuvant chemotherapy**				
No	Ref		Ref	
Yes	1.72 (1.36-2.16)	**<0.001**	0.86 (0.67-1.10)	0.22
**Postoperative complication**				
No	Ref			
Yes	1.08 (0.72-1.60)	0.72		
**Anti – *H pylori* treatment**				
No	Ref		Ref	
Yes	0.29 (0.17-0.50)	**<0.001**	0.48 (0.28-0.83)	**0.008**

Abbreviations: HR: hazard ratio; BMI: Body mass index; CI: confidence interval; Ref: Reference
The variables with statistical significance in the univariable analysis were entered into the multivariable Cox regression analysis, such as Age group, diabetes, TNM stage, tumor maximum diameter, adjuvant chemotherapy and anti–H pylori treatment.

© 2024 Zhao Z et al. *JAMA Network Open.*

eFigure 1. Subgroup Analyses for Overall Survival According to Clinical Indicators Including Age Group, Sex, Tumor Differentiation, etc

Characteristics	Anti–*H pylori* treatment n(%)	Non–anti–*H pylori* treatment n(%)	HR(95% CI)	P Value
Overall(N=1293)	125	1168	0.33 (0.18,0.60)	<0.001
**Sex**				
Male(n=860)	85 (68.0%)	775 (66.4%)	0.36 (0.18,0.70)	0.003
Female(n=433)	40 (32.0%)	393 (33.6%)	0.23 (0.06,0.93)	0.04
**Age group, y**				
<60(n=697)	75 (60.0%)	622 (53.3%)	0.30 (0.12,0.74)	0.009
≥60(n=596)	50 (40.0%)	546 (46.7%)	0.36 (0.16,0.81)	0.01
**Classification of BMI**				
Normal weight(n=778)	78 (62.4%)	700 (59.9%)	0.46 (0.24,0.91)	0.03
Abnormal weight(n=515)	47 (37.6%)	468 (40.1%)	0.14 (0.04,0.58)	0.007
**History of smoking**				
No(n=824)	81 (64.8%)	743 (63.6%)	0.29 (0.13,0.65)	0.003
Yes(n=469)	44 (35.2%)	425 (36.4%)	0.40 (0.16,0.99)	<0.05
**Hypertension**				
No(n=1114)	109 (87.2%)	1005 (86.0%)	0.30 (0.16,0.59)	<0.001
Yes(n=179)	16 (12.8%)	163 (14.0%)	0.53 (0.13,2.21)	0.38
**Coronary heart disease**				
No(n=1279)	124 (99.2%)	1155 (98.9%)	0.33 (0.18,0.61)	<0.001
Yes(n=14)	1 (0.8%)	13 (1.1%)	ns	ns
**Diabetes**				
No(n=1205)	119 (95.2%)	1086 (93.0%)	0.32 (0.17,0.61)	<0.001
Yes(n=88)	6 (4.8%)	82 (7.0%)	0.37 (0.05,2.78)	0.33
**Histologic grade**				
G1/G2(n=461)	48 (38.4%)	413 (35.4%)	0.33 (0.12,0.89)	0.03
G3/G4(n=752)	69 (55.2%)	683 (58.5%)	0.30 (0.13,0.69)	0.004
Others(n=80)	8 (6.4%)	72 (6.2%)	0.71 (0.09,5.51)	0.74
**Borrmann classification**				
IV(n=38)	4 (3.2%)	34 (2.9%)	1.69 (0.38,7.45)	0.49
I–III(n=1233)	116 (92.8%)	1117 (95.6%)	0.26 (0.13,0.53)	<0.001
Unknown(n=22)	5 (4.0%)	17 (1.5%)	1.65 (0.15,18.25)	0.68
**Lauren classification**				
Diffused(n=495)	50 (40.0%)	445 (38.1%)	0.06 (0.01,0.43)	0.005
Intestinal(n=359)	31 (24.8%)	328 (28.1%)	0.33 (0.08,1.38)	0.13
Mixed(n=298)	28 (22.4%)	270 (23.1%)	0.60 (0.19,1.95)	0.40
Unknown(n=141)	16 (12.8%)	125 (10.7%)	0.80 (0.32,2.01)	0.63
**Pathological T stage**				
T1/T2(n=464)	82 (65.6%)	382 (32.7%)	0.56 (0.17,1.88)	0.35
T3/T4(n=829)	43 (34.4%)	786 (67.3%)	0.47 (0.23,0.94)	0.03
**Pathological N stage**				
N−(n=471)	75 (60.0%)	396 (33.9%)	0.45 (0.14,1.46)	0.18
N+(n=822)	50 (40.0%)	772 (66.1%)	0.43 (0.21,0.86)	0.02
**TNM stage**				
I(n=350)	70 (56.0%)	280 (24.0%)	0.86 (0.19,4.00)	0.85
II/III(n=943)	55 (44.0%)	888 (76.0%)	0.43 (0.22,0.85)	0.01
**Tumor maximum diameter, cm**				
≤4(n=720)	99 (79.2%)	621 (53.2%)	0.30 (0.11,0.82)	0.02
>4(n=573)	26 (20.8%)	547 (46.8%)	0.63 (0.29,1.34)	0.23
**Type of gastrectomy**				
Distal(n=829)	102 (81.6%)	727 (62.2%)	0.31 (0.14,0.71)	0.006
Proximal(n=140)	11 (8.8%)	129 (11.0%)	0.25 (0.03,1.84)	0.17
Total(n=324)	12 (9.6%)	312 (26.7%)	0.77 (0.28,2.11)	0.61
**CEA, ng/ml**				
≤5(n=1083)	112 (89.6%)	971 (83.1%)	0.30 (0.15,0.62)	<0.001
>5(n=210)	13 (10.4%)	197 (16.9%)	0.57 (0.18,1.80)	0.33
**Adjuvant chemotherapy**				
No(n=485)	71 (56.8%)	414 (35.4%)	0.20 (0.06,0.62)	0.006
Yes(n=808)	54 (43.2%)	754 (64.6%)	0.48 (0.24,0.97)	0.04
**Postoperative complication**				
No(n=1214)	116 (92.8%)	1098 (94.0%)	0.33 (0.17,0.62)	<0.001
Yes(n=79)	9 (7.2%)	70 (6.0%)	0.36 (0.05,2.74)	0.33

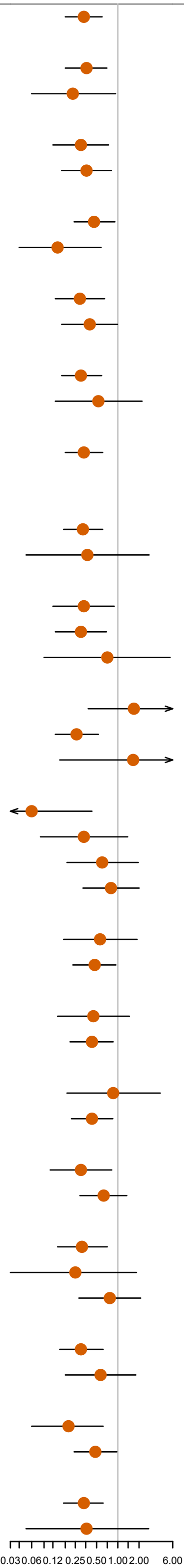

Favors anti–*H pylori* treatment | Favors non–anti–*H pylori* treatment

© 2024 Zhao Z et al. *JAMA Network Open.*

eFigure 2. Subgroup Analyses for Disease-Free Survival According to Clinical Indicators Including Age Group, Sex, Tumor Differentiation, etc

Characteristics	Anti–*H pylori* treatment n(%)	Non–anti–*H pylori* treatment n(%)	HR(95% CI)	P Value
Overall(N=1293)	125	1168	0.29 (0.17,0.50)	<0.001
**Sex**				
Male(n=860)	85 (68.0%)	775 (66.4%)	0.34 (0.19,0.61)	<0.001
Female(n=433)	40 (32.0%)	393 (33.6%)	0.15 (0.04,0.61)	0.008
**Age group, y**				
<60(n=697)	75 (60.0%)	622 (53.3%)	0.31 (0.15,0.64)	0.001
≥60(n=596)	50 (40.0%)	546 (46.7%)	0.28 (0.12,0.62)	0.002
**Classification of BMI**				
Normal weight(n=778)	78 (62.4%)	700 (59.9%)	0.37 (0.20,0.71)	0.002
Abnormal weight(n=515)	47 (37.6%)	468 (40.1%)	0.19 (0.07,0.50)	<0.001
**History of smoking**				
No(n=824)	81 (64.8%)	743 (63.6%)	0.24 (0.11,0.50)	<0.001
Yes(n=469)	44 (35.2%)	425 (36.4%)	0.39 (0.18,0.84)	0.02
**Hypertension**				
No(n=1114)	109 (87.2%)	1005 (86.0%)	0.28 (0.16,0.51)	<0.001
Yes(n=179)	16 (12.8%)	163 (14.0%)	0.34 (0.08,1.38)	0.13
**Coronary heart disease**				
No(n=1279)	124 (99.2%)	1155 (98.9%)	0.29 (0.17,0.50)	<0.001
Yes(n=14)	1 (0.8%)	13 (1.1%)	ns	ns
**Diabetes**				
No(n=1205)	119 (95.2%)	1086 (93.0%)	0.27 (0.15,0.48)	<0.001
Yes(n=88)	6 (4.8%)	82 (7.0%)	0.54 (0.13,2.27)	0.40
**Histologic grade**				
G1/G2(n=461)	48 (38.4%)	413 (35.4%)	0.34 (0.14,0.82)	0.02
G3/G4(n=752)	69 (55.2%)	683 (58.5%)	0.26 (0.13,0.53)	<0.001
Others(n=80)	8 (6.4%)	72 (6.2%)	0.34 (0.05,2.54)	0.29
**Borrmann classification**				
IV(n=38)	4 (3.2%)	34 (2.9%)	1.14 (0.26,4.92)	0.86
I–III(n=1233)	116 (92.8%)	1117 (95.6%)	0.25 (0.14,0.45)	<0.001
Unknown(n=22)	5 (4.0%)	17 (1.5%)	0.61 (0.07,5.52)	0.66
**Lauren classification**				
Diffused(n=495)	50 (40.0%)	445 (38.1%)	0.12 (0.04,0.38)	<0.001
Intestinal(n=359)	31 (24.8%)	328 (28.1%)	0.33 (0.10,1.03)	0.06
Mixed(n=298)	28 (22.4%)	270 (23.1%)	0.35 (0.11,1.10)	0.07
Unknown(n=141)	16 (12.8%)	125 (10.7%)	0.75 (0.30,1.88)	0.54
**Pathological T stage**				
T1/T2(n=464)	82 (65.6%)	382 (32.7%)	0.41 (0.14,1.13)	0.09
T3/T4(n=829)	43 (34.4%)	786 (67.3%)	0.46 (0.24,0.86)	0.02
**Pathological N stage**				
N−(n=471)	75 (60.0%)	396 (33.9%)	0.39 (0.14,1.10)	0.07
N+(n=822)	50 (40.0%)	772 (66.1%)	0.37 (0.20,0.70)	0.002
**TNM stage**				
I(n=350)	70 (56.0%)	280 (24.0%)	0.62 (0.18,2.11)	0.44
II/III(n=943)	55 (44.0%)	888 (76.0%)	0.39 (0.21,0.71)	0.002
**Tumor maximum diameter, cm**				
≤4(n=720)	99 (79.2%)	621 (53.2%)	0.31 (0.14,0.70)	0.005
>4(n=573)	26 (20.8%)	547 (46.8%)	0.50 (0.25,1.01)	0.05
**Type of gastrectomy**				
Distal(n=829)	102 (81.6%)	727 (62.2%)	0.27 (0.13,0.56)	<0.001
Proximal(n=140)	11 (8.8%)	129 (11.0%)	0.31 (0.08,1.29)	0.11
Total(n=324)	12 (9.6%)	312 (26.7%)	0.63 (0.23,1.70)	0.36
**CEA, ng/ml**				
≤5(n=1083)	112 (89.6%)	971 (83.1%)	0.28 (0.15,0.50)	<0.001
>5(n=210)	13 (10.4%)	197 (16.9%)	0.49 (0.15,1.54)	0.22
**Adjuvant chemotherapy**				
No(n=485)	71 (56.8%)	414 (35.4%)	0.20 (0.07,0.55)	0.002
Yes(n=808)	54 (43.2%)	754 (64.6%)	0.40 (0.21,0.76)	0.005
**Postoperative complication**				
No(n=1214)	116 (92.8%)	1098 (94.0%)	0.27 (0.15,0.49)	<0.001
Yes(n=79)	9 (7.2%)	70 (6.0%)	0.49 (0.12,2.09)	0.34

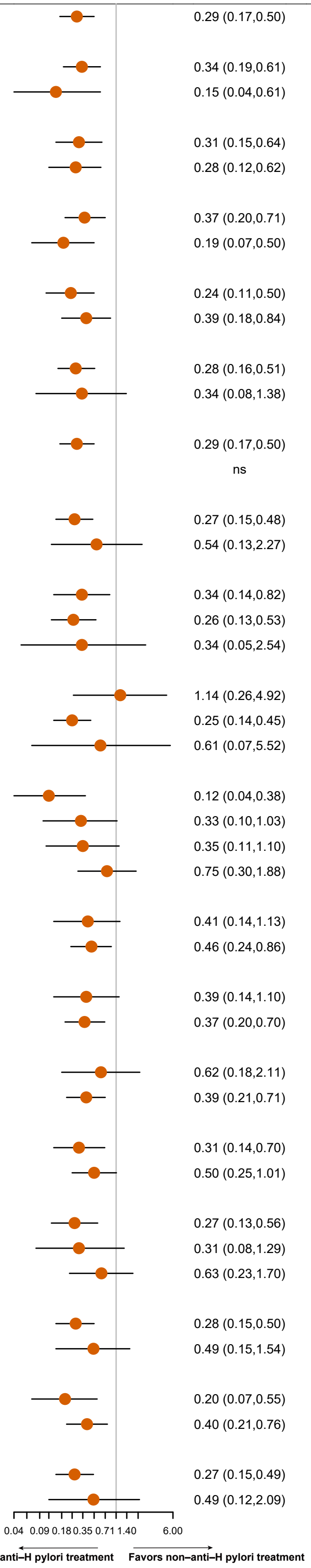

© 2024 Zhao Z et al. *JAMA Network Open.*
