# Supplementary material for: Anti–Helicobacter pylori Treatment in Patients With Gastric Cancer After Radical Gastrectomy
Source: JAMA Netw Open. 2024 Mar 28;7(3):e243812. doi: 10.1001/jamanetworkopen.2024.3812 (PMC10979314; doi:10.1001/jamanetworkopen.2024.3812)
Supplement: Supplement 2. — Data Sharing Statement [file jamanetwopen-e243812-s002.pdf]

Data Sharing Statement

Zhao. Anti–Helicobacter pylori Treatment in Patients With Gastric Cancer After Radical Gastrectomy. *JAMA Netw Open*. Published March 27, 2024.
doi:10.1001/jamanetworkopen.2024.3812

Data
Data available: Yes
Data types: Deidentified participant data, Data dictionary
How to access data: nierc@sysucc.org.cn
When available: With publication

Supporting Documents
Document types: Statistical/analytic code
How to access documents: nierc@sysucc.org.cn
When available: With publication

Additional Information
Who can access the data: researchers whose proposed use of the data has been approved.
Types of analyses: The data sharing request for legitimate purposes will be met.
Mechanisms of data availability: after approval of a proposal, and with a signed data access agreement.
